# Uptake of environmental halophilic archaea by human dendritic cells

**DOI:** 10.1038/s41598-025-07365-z

**Published:** 2025-07-01

**Authors:** Krzysztof Krawczyk, Dorota Rybaczek, Camille Locht, Agata Pyrzanowska-Banasiak, Martyna Ciołek, Paulina Sicinska, Michalina Rachubik, Magdalena Kowalewicz-Kulbat

**Affiliations:** 1 Department of Immunology and Infectious Biology, Institute of Microbiology, Biotechnology and Immunology, Faculty of Biology and Environmental Protection, University of Lodz, 90-237 Lodz, Poland; 2 Department of Cytophysiology, Institute of Experimental Biology, Faculty of Biology and Environmental Protection, University of Lodz, Pomorska 141/143, 90-236 Lodz, Poland; 3 Univ. Lille, CNRS, Inserm, CHU Lille, Institut Pasteur de Lille, U1019-UMR9017-CIIL-Center for Infection and Immunity of Lille, 59000 Lille, France; 4 Department of Biophysics of Environmental Pollution, Faculty of Biology and Environmental Protection, University of Lodz, Pomorska Str. 141/143, 90-236 Lodz, Poland

**Keywords:** Halophilic archaea, Dendritic cells, SEB, DNA breaks, Apoptosis, Cell cycle, Microbiology, Archaea, Immunology, Innate immune cells

## Abstract

Halophilic archaea are a group distinct from Bacteria and Eukarya, which belong to extremophiles living in highly saline environments. However, they can also exist in the human microbiome. Their impact on the human immune system is poorly known. In this study we examined the interaction of *Halorhabdus rudnickae* WSM-64^T^, isolated from the Barycz area of the Wieliczka Salt-Mine in Poland, and of *Natrinema salaciae* MDB25^T^ from the brine of Lake Medee in Italy, with human monocyte-derived dendritic cells (Mo-DCs). We found that these halophilic archaea invade the cytoplasm and the nucleus of Mo-DCs, but, in contrast to intracellular bacterial pathogens, they do not cause cytotoxic effects on DCs, as no single- or double-stranded DNA breaks (SSB and DSB, respectively), nor chromatin aberrations were noted. Moreover, they did not induce cell cycle alterations, apoptosis or necrosis of DCs. Surprisingly, these halophiles were found to protect against genotoxic activities of *Staphylococcus aureus* enterotoxin B (SEB), as pre-incubation of the Mo-DCs with the halophilic archaea significantly reduced SEB-induced SSB and DSB, as well as cell cycle disturbance and apoptosis. Therefore, these halophilic archaea can be regarded as safe stimulators for the Mo-DCs to potentially be used as immunomodulators and protective agents for various disorders.

**Supplementary Information:**

The online version contains supplementary material available at 10.1038/s41598-025-07365-z.

## Introduction

Archaea are mostly extremophiles, living in conditions of high salinity, low or high temperatures, pressure, extreme pH or in the presence of heavy metals^1^. Exposure of humans to extremophilic, especially halophilic archaea may occur in salt-mines during halotherapy sessions, which are used to treat respiratory conditions, such as asthma, cystic fibrosis, but also dermatitis and others^2^. The interactions of halophilic archaea with the human immune system still remains poorly known.

Dendritic cells (DCs) are the prime innate immune cells that recognize antigens and present them to T cells to induce adaptive immune responses^3^. They also control the responses of T cells by delivering co-stimulatory signals and cytokines^4^. Studies on the intestinal methanogenic archaea *Methanosphaera stadtmanae* have shown that the human immune system recognizes this microorganism via TLR-7 and TLR-8, leading to activation of the NLRP3 inflammasome^5^ and to enhanced production of cytokines (TNF-alpha and IL-1-beta) and expression of the surface markers CD86 and CD197^6^. Our previous study on the environmental halophilic archaea *Halorhabdus rudnickae* and *Natrinema salaciae* showed that they can stimulate monocyte-derived DCs (Mo-DCs) to increase their expression of CD86, CD80 and CD83 and to produce IL-10, IL-12p40 and TNF-alpha. Moreover, autologous CD4^+^ T cells co-cultured with halophile-stimulated Mo-DCs produced significantly more IFN-gamma and IL-13 compared to T cells co-cultured with unstimulated DCs^7^.

In this study we further examined the interaction of *Hrd. rudnickae* WSM-64^T^ (= DSM 29498^T^ = CECT 8673^T^), isolated from the Barycz mining area of the Wieliczka Salt-Mine in Poland^8^, and of *Nnm. salaciae* MDB25^T^ (= DSM 25055^T^ = JCM 17869^T^), isolated from the brine of Lake Medee in Italy^9^, with human Mo-DCs. We found that these halophilic archaea are able to invade the cytoplasm and the nucleus of DCs, but do not cause cell death or DNA damage, nor do they interfere with Mo-DC cell cycle. Instead, they provided protection against genotoxic activities of *Staphylococcus aureus* enterotoxin B (SEB).

## Results

### Halophilic archaea invade dendritic cells

**Fig. 1:**
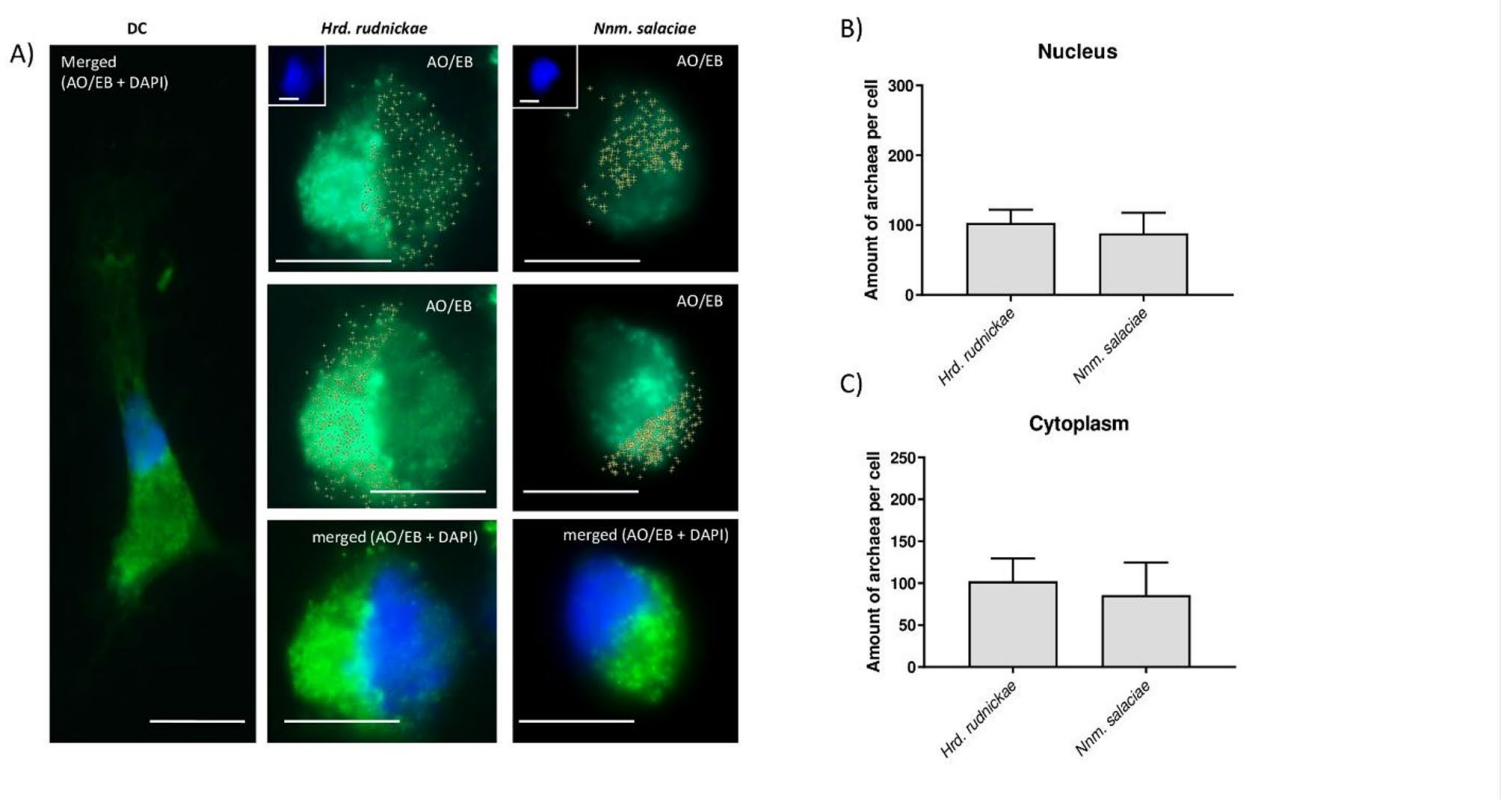
Presence of halophilic archaea in the cytoplasm and nucleus of DCs. (**A**) Representative microscopic images showing the presence *Hrd. rudnickae* or *Nnm. salaciae* within the nucleus and cytoplasm of DCs. Crosses indicate halophilic archaea, the blue color the nucleus and the light green color the cytoplasm. AO, acridine orange; EB, ethidium bromide; DC, unstimulated DCs. Scale bars are 10 μm. The numbers of halophilic archaea (*Hrd. rudnickae* or *Nnm. salaciae*) in the nucleus (**B**) and cytoplasm (**C**) of DCs are expressed as means ± standard deviation of a minimum of 100 counts for each experiment in 4 independent experiments.

To determine the fate of the halophilic archaea *Hrd. rudnickae* and *Nnm. salaciae* brought in contact with DCs, the cells were incubated with the archaea for 24 h and then analyzed by fluorescence microscopy after staining with DAPI, ethidium bromide (EB) and acridine orange (AO). Preliminary examination using AO and EB staining to quantify dead, dying and living archaeal cells^10^ (Fig. S1A) indicated that approximately 90% and 70% of *Hrd. rudnickae* and *Nnm. salaciae*, respectively, survived for at least one hour in DC culture medium (Fig. S1B), and approximately up to 70% of each strain was still alive after 30 days of incubation in this medium (Fig. S1C). After 24 h co-culture with DCs, numerous halophilic archaea, both *Hrd. rudnickae* and *Nnm. salaciae*, could be detected within the cytoplasm, as well as within the cell nucleus of the Mo-DCs (Fig. 1A). To quantify the amounts of the halophilic archaea within the cytoplasm and the nucleus, DCs were analyzed by using the ImageJ v1.37c software, and the average numbers of halophilic archaea present in the cell nucleus and cytoplasm were determined. As shown in Fig. 1B and C, an average of approximately 100 *Hrd. rudnickae* or *Nnm. salaciae* organisms per cell were found in the cytoplasm and nucleus of the Mo-DCs. There were no statistical differences between the two halophilic strains concerning the amounts of halophilic archaea within the cytoplasm and nucleus of DCs.

To confirm that DCs can uptake the halophilic archaeal strains *Hrd. rudnickae* and *Nnm. salaciae* we incubated them for 24 h in the presence of either strain. After extensive washing to remove the extracellular halophiles, polymerase chain reactions (PCR) were performed on the lysed cells to amplify the 16 S rRNA gene using halophilic archaeal-specific primers. As expected, no DNA was amplified with DC lysates that had not been incubated with the halophiles, used as a negative control. In contrast, 16 S rRNA DNA could readily be amplified from lysates of DC incubated with either halophile (Fig. S2).

### Halophilic archaea do not change mitotic, nor aberration index within DCs

**Fig. 2:**
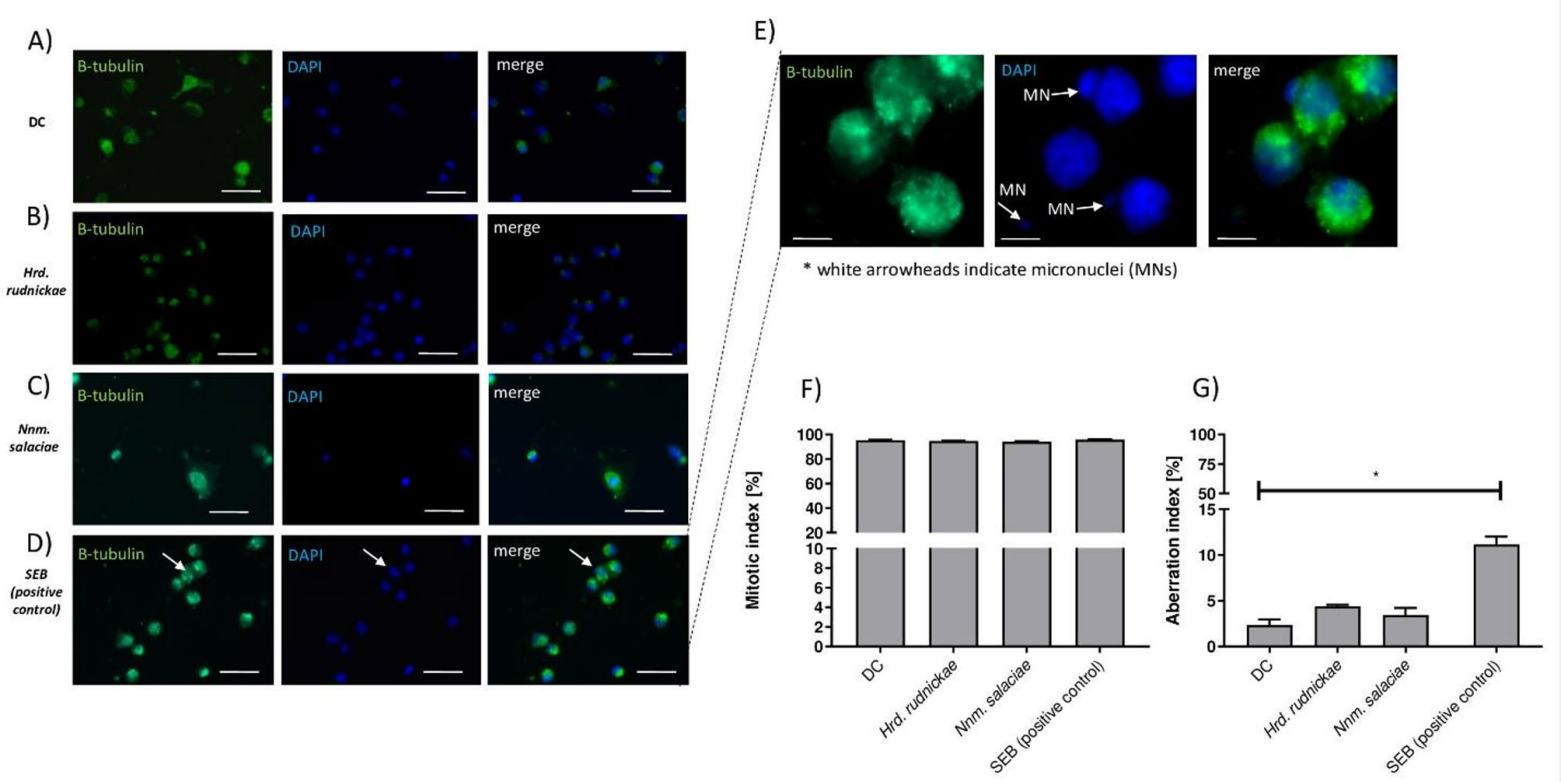
Lack of chromosome aberration in DCs after incubation with halophilic archaea. Representative microscopic images showing unstimulated DCs, used as negative controls (**A**), DCs incubated with *Hrd. rudnickae* (**B**), *Nnm. salaciae* (**C**) or stimulated with SEB, used as a positive control (**D**). Magnified image of SEB-stimulated DCs with chromosome aberrations (**E**). MN, micronuclei. Scale bars are 10 μm. Mitotic index (**F**) and aberration index (**G**) of unstimulated DCs, DCs incubated with *Hrd. rudnickae*, *Nnm. salaciae* or stimulated with SEB are presented as means ± standard deviation. *p < 0.05; *n* = 4; *DC* unstimulated DCs.

As the halophilic archaea *Hrd. rudnickae* and *Nnm. salaciae* are able to invade the cytoplasm and the nucleus of DCs, we wondered whether this may have an effect on the cellular DNA. We therefore determined whether incubation of the DCs with these halophilic archaea resulted in changes in chromatin condensation, as evaluated by the mitotic index, or chromatin fragmentation, as evaluated by the aberration index of the DCs. Human Mo-DCs were incubated with each of these halophilic archaea for 24 h, stained with DAPI and analyzed by fluorescence microscopy (Fig. 2A–E). DCs in the absence of halophiles and DCs incubated with SEB served as negative and positive controls, respectively. In addition, monoclonal antibodies against human β-tubulin were used to analyze the DC cytoskeleton (Fig. S3). Chromatin fragmentation was assessed by fluorescence of the nucleus using DAPI staining, with lower fluorescence indicating increased chromatin fragmentation. Lower levels of fluorescence also indicated less chromatin condensation. After 24 h incubation of DCs with the halophilic archaea, no significant changes were observed in the degree of chromatin condensation (Fig. 2F), nor in the percentage of chromatin fragmentation within the DCs compared to cells incubated in the absence of the halophilic archaea (Fig. 2G). In contrast, while DC stimulation with SEB did not result in significant changes in the level of DC chromatin condensation compared to unstimulated cells (Fig. 2F), a significantly higher percentage of chromatin fragmentation was observed in SEB-stimulated DCs compared to unstimulated cells (*p* = 0.0159) (Fig. 2D,E,G).

### Halophilic archaea do not induce DNA breakage of DCs

**Fig. 3:**
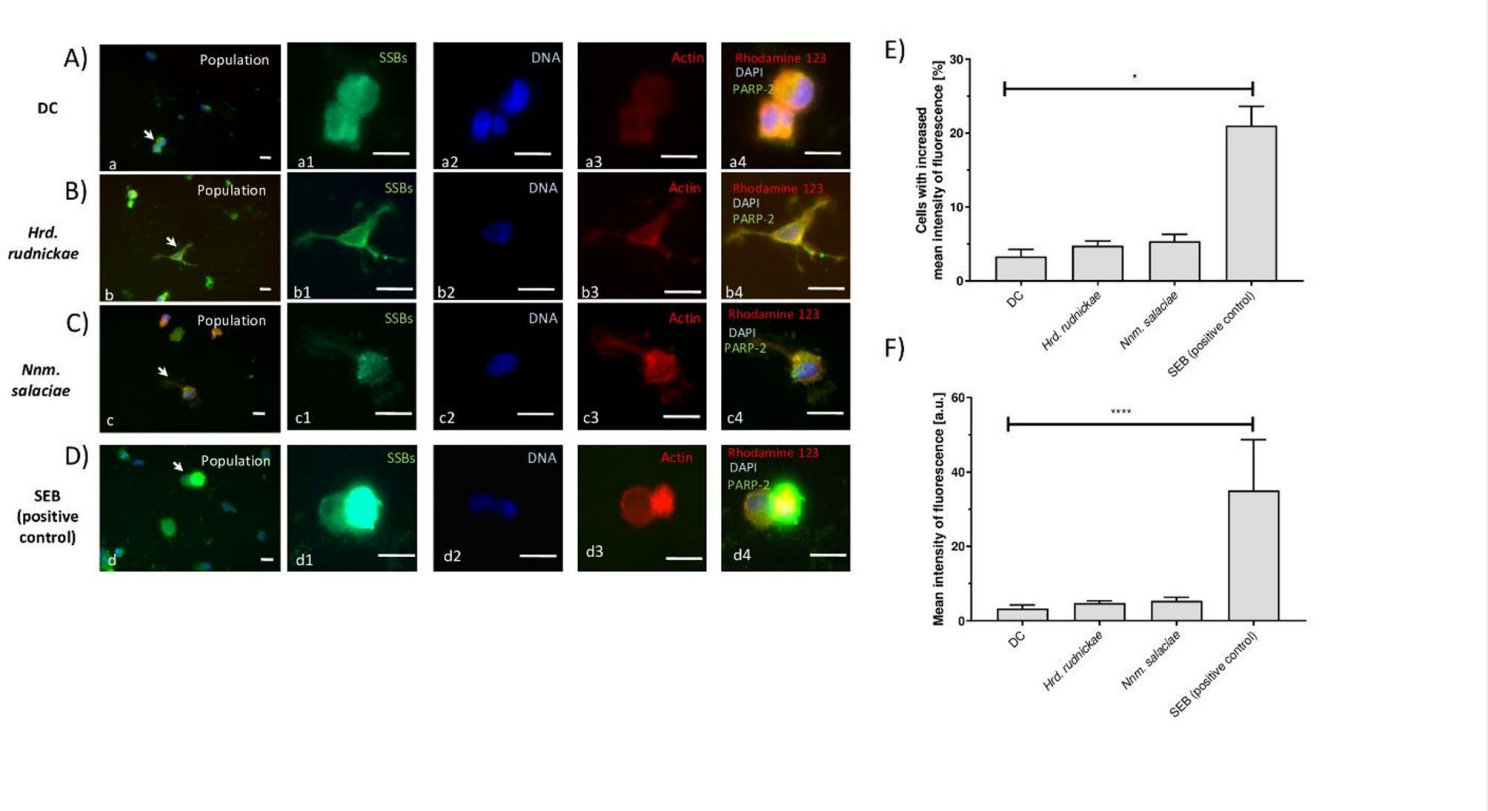
Lack of single-stranded DNA breaks in DCs after incubation with halophilic archaea. Representative microscopic images showing SSBs in DCs. DC shows unstimulated DCs, used as a negative control (**A**), *Hrd. rudnickae* (**B**) and *Nnm. salaciae* (**C**) show DCs incubated with the respective halophilic archaea and SEB (**D**) shows DCs stimulated with SEB, used as a positive control. Panels 1 show SSBs using PARP-2 staining. Panels 2 show DAPI staining of DNA. Panels 3 show actin staining with Rhodamine 123, and panels 4 show merged images of all three. Scale bars are 10 μm. Percentages of cells with increased mean intensity of fluorescence indicating single stranded DNA breaks (**E**) and mean intensity of fluorescence in single cell in arbitrary units (**F**) of DC unstimulated, incubated with *Hrd. rudnickae*, *Nnm. salaciae* or stimulated with SEB are presented as means ± standard deviation based on a minimum of 8 counts of 4 independent experiments. *p < 0.05; ****p ≤ 0.0001.

To further investigate the effect of halophilic archaea on the DNA within DCs, single-stranded breaks (SSB) and double-stranded breaks (DSB) were analyzed. DCs were incubated with *Hrd. rudnickae* or *Nnm. salaciae* for 24 h and then stained with monoclonal antibodies directed against PARP-2, an enzyme that binds to SSB in DNA, which thereby can be used to identify DNA containing SSB (Fig. 3A–D). Again, DCs in the absence of the halophilic archaea and SEB-stimulated DCs were used as negative and positive controls, respectively (Figs. S4 and S5). Representative microscopic images showing DCs with SSB are shown in Fig. 3D. Incubation of DCs with *Hrd. rudnickae* or *Nnm. salaciae* did not significantly increase the percentage of cells with SSBs (Fig. 3E), nor the number of SSBs within a single DC (Fig. 3F). In contrast, SEB-stimulated DCs, used as a positive control, showed a significantly higher percentage of cells with SSBs (*p* = 0.0286) (Fig. 3E), as well as significantly higher numbers of SSBs per cell (*p* < 0.0001) compared to unstimulated DCs (Fig. 3F).

**Fig. 4:**
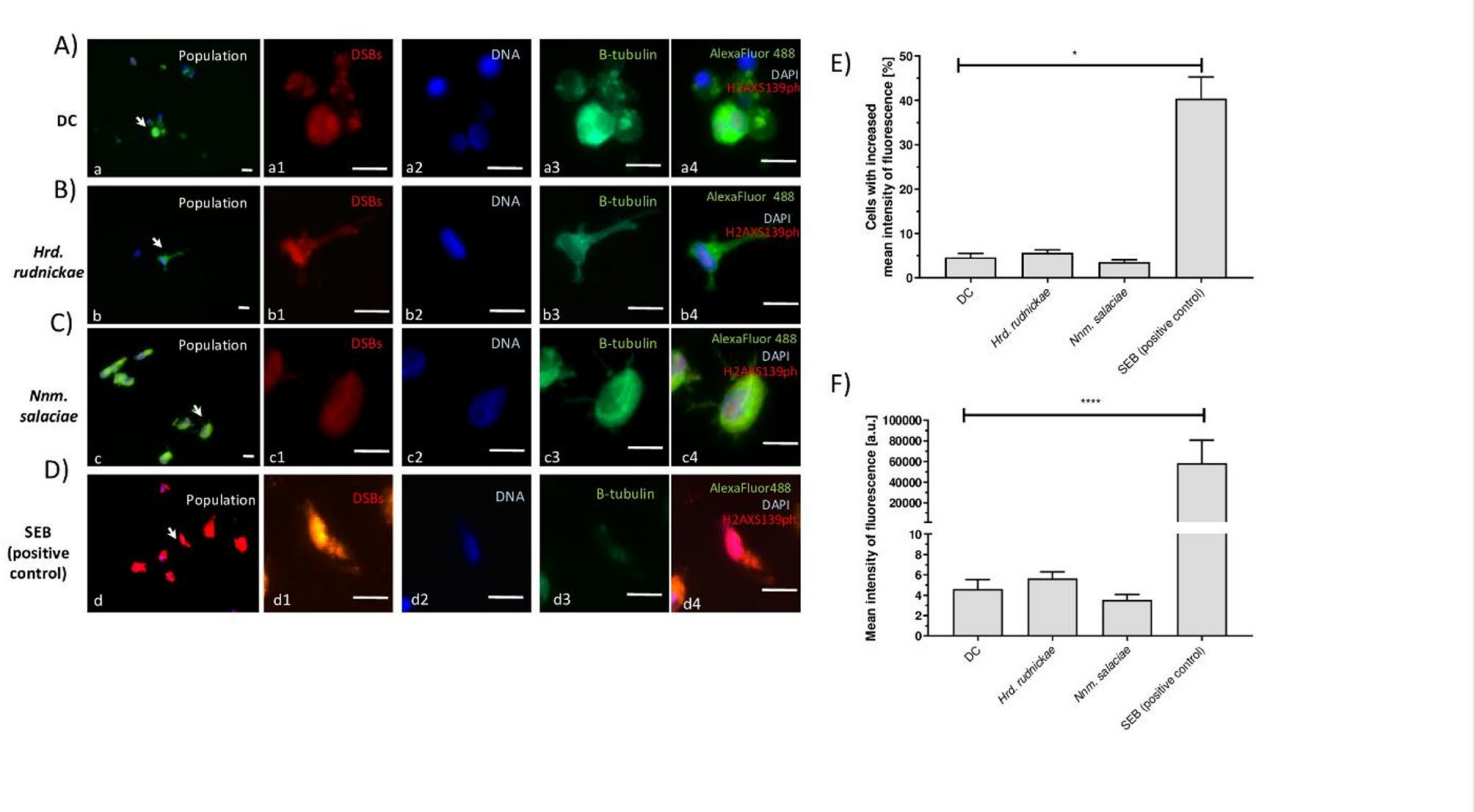
Lack of double-stranded DNA breaks in DCs after incubation with halophilic archaea. Representative microscopic images showing DSB in DCs. DC represents unstimulated DCs, used as negative controls (**A**), *Hrd. rudnickae* (**B**) and *Nnm. salaciae* (**C**) show DCs incubated with the respective strains and SEB (**D**) shows DCs stimulated with SEB, used as a positive control. Scale bars are 10 μm. Panels 1 show DSBs by H2AXS139ph staining, panels 2 show DAPI staining of DNA, panels 3 show β-tubulin staining, and panels 4 show merged images of all three. Percentages of cells with increased mean intensity of fluorescence indicating DSB (**E**) and mean intensity of fluorescence in single cell in arbitrary units (**F**) in the different conditions are presented as means ± standard deviation based on a minimum of 8 counts of 4 independent experiments. *p < 0.05; ****p ≤ 0.0001;

To determine the effect of the halophilic archaea on DSB formation, the attachment sites of phospho-H2AX to DNA were visualized by fluorescence microscopy using fluorochrome-conjugated monoclonal antibodies against phospho-H2AX to detect double-stranded DNA damage after incubation of the DCs with *Hrd. rudnickae* or *Nnm. salaciae* for 24 h. As shown in the representative microscopic images (Fig. 4A-D), incubation of the DCs with *Hrd. rudnickae* or *Nnm. salaciae* did not significantly increase the percentage of cells with DSB (Fig. 4E), nor the number of DSBs per cell (Fig. 4F), in contrast to SEB-stimulated DCs, which showed a significantly higher percentage of cells with DSBs (*p* = 0.0286) (Fig. 4E), as well as significantly more DSBs per cell (*p* < 0.0001) compared to unstimulated DCs (Fig. 4F).

### Halophilic archaea do not modify the cell cycle of DCs

**Fig. 5:**
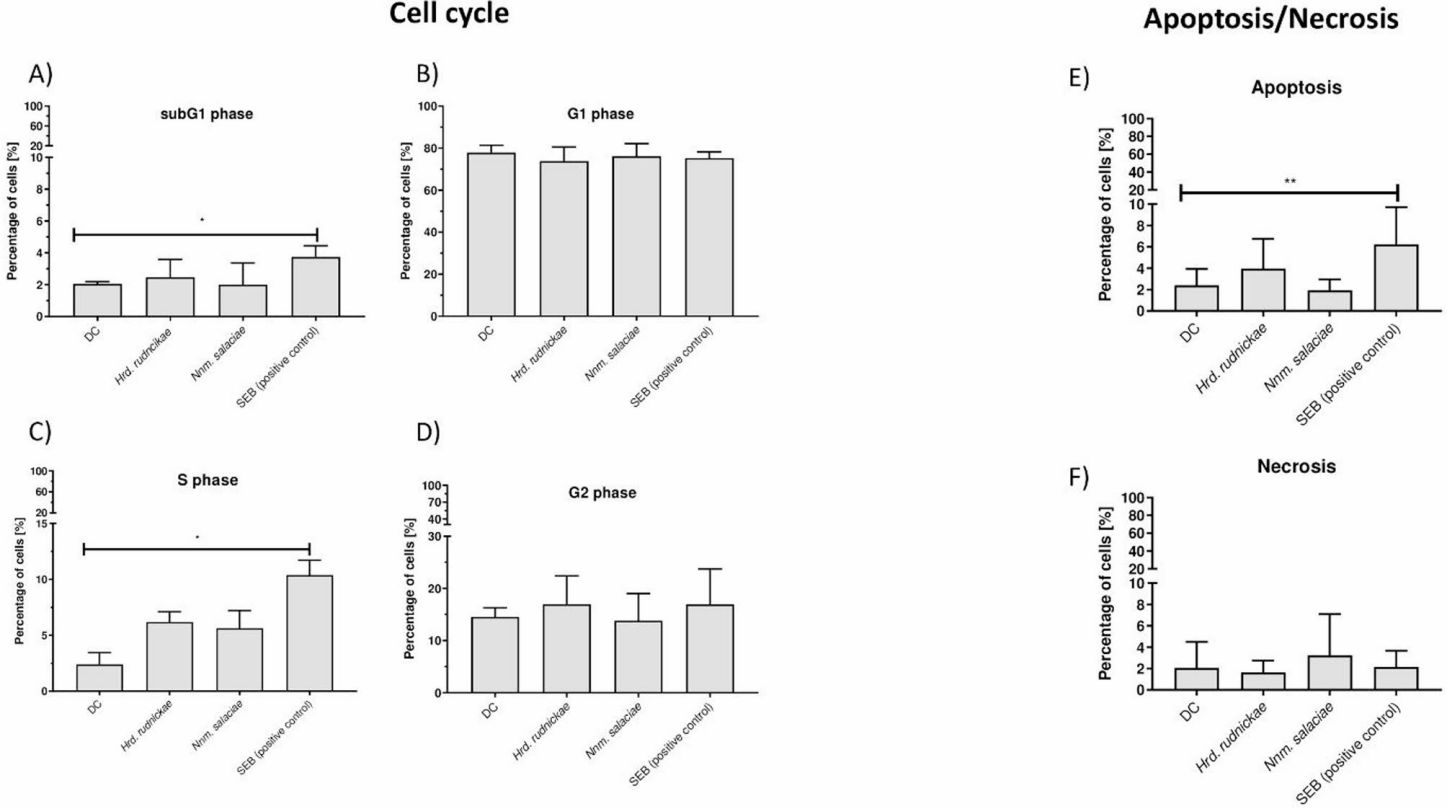
Lack of cell cycle disturbances in DCs after incubation with halophilic archaea. Percentages of unstimulated DCs, DCs incubated with *Hrd. rudnickae*, *Nnm. salaciae* or stimulated with SEB in the cell cycle subG1 (**A**), G1 (**B**), S (**C**) and G2 phases (**D**), assessed by flow cytometry, and percentages of unstimulated DCs, DCs incubated with *Hrd. rudnickae*, *Nnm. salaciae* or stimulated SEB undergoing apoptosis (**E**) or necrosis (F) are presented as means ± standard deviation from seven independent experiments. *p ≤ 0.05; **p ≤ 0.01.

The impact of halophilic archaea on the DC cell cycle was also investigated. The cell cycle of human Mo-DCs incubated with halophilic archaea for 24 h were analyzed by flow cytometry upon staining with propidium iodide, which allowed us to quantify the DNA content of the cells at specific phases of the cell cycle. As shown in Fig. 5, the percentages of the DCs at different cell cycle stages did not significantly change upon incubation with either of the two halophilic strains, indicating that these halophiles had no significant impact on the cell cycle changes in the sub-G1, G1, S and G2 phases, while SEB stimulation of the DCs resulted in a significant increase in cell accumulation in the sub-G1 (*p* = 0.0286) (Fig. 5A) and S phases (*p* = 0.0286) (Fig. 5C).

### Halophilic archaea do not induce apoptosis nor necrosis of DCs

Finally, we examined the effect of the halophilic archaea *Hrd. rudnickae* and *Nnm. salaciae* on apoptosis and necrosis of DCs. Upon incubation with these halophilic archaea for 24 h the percentage of DCs undergoing apoptosis was assessed by flow cytometry using the FITC-labelled annexin V kit and propidium iodide. By using both markers, it is possible to distinguish three cell populations: living cells (not staining with either compound), necrotic cells (staining with propidium iodide), and apoptotic cells (staining with annexin V and propidium iodide)^11^. Propidium iodide enters the cell through the damaged cell membrane and then binds to nucleic acids, while annexin V specifically binds to phosphatidylserine, which moves from the inner to the outer side of the cell membrane in cells undergoing apoptosis.

No significant increase in the percentage of cells undergoing apoptosis (Fig. 5E) or necrosis (Fig. 5F) was observed following incubation of DCs with the halophilic archaea, whereas stimulation of DCs with the SEB resulted in a significant increase in the percentage of cells undergoing apoptosis (*p* = 0.0062) (Fig. 5E), but not of necrosis (Fig. 5F).

### Halophilic archaea protect DCs from genotoxic effects of SEB

**Fig. 6:**
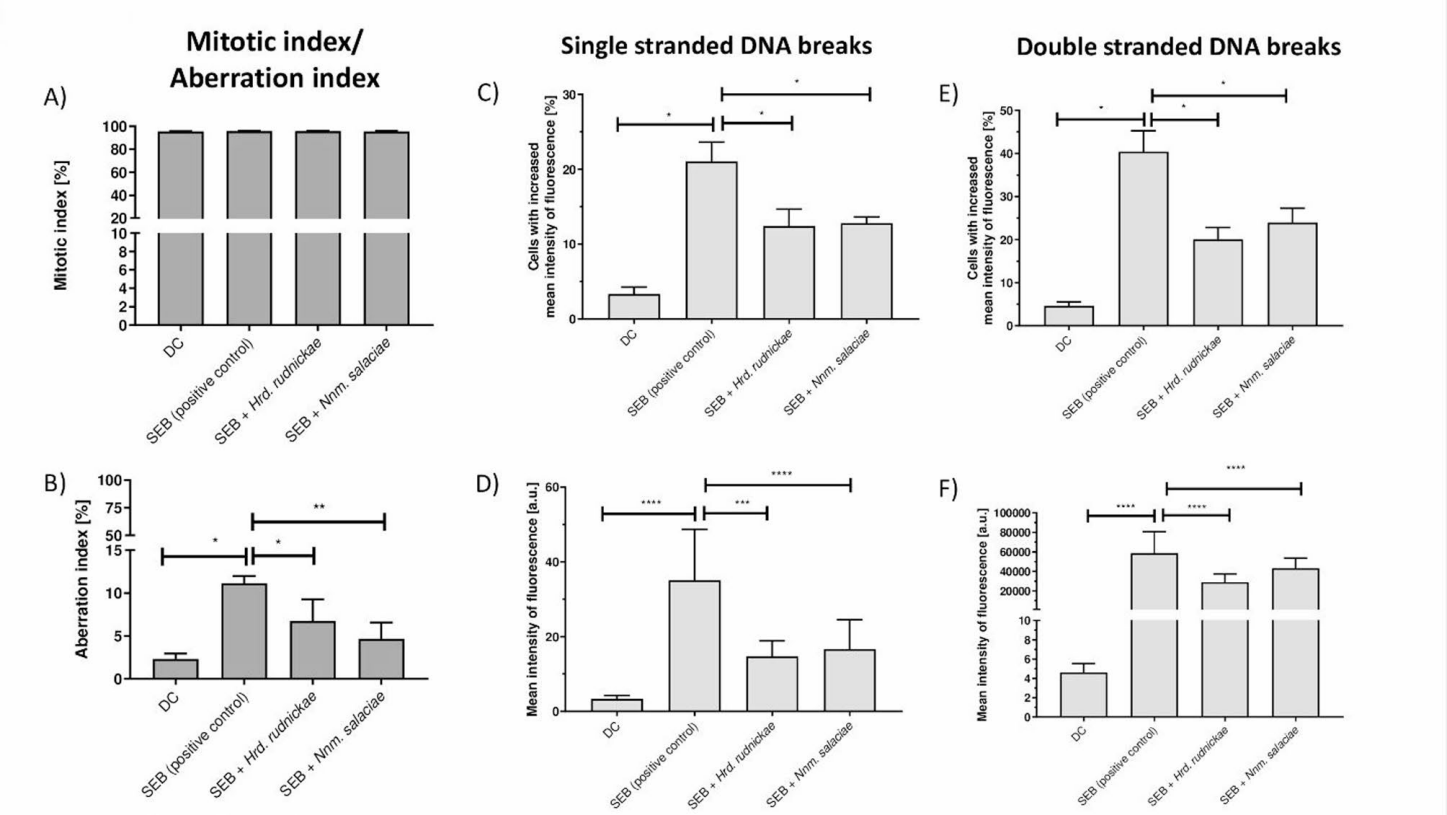
Protection by halophilic archaea against SEB-induced double- and single-stranded DNA breaks. Mitotic index (**A**), aberration index (**B**) SSB (**C**,**D**) and DSB (**E**,**F**) of unstimulated DCs, used as negative controls, incubated with *Hrd. rudnickae* + SEB, *Nnm. salaciae* + SEB or stimulated with SEB alone are presented as means ± SD based on 4 (panels **A** and **B**) and a minimum of 20 counts in 4 different experiments. *p < 0.05; ***p ≤ 0.001; ****p ≤ 0.0001. The percentages of cells with increased mean fluorescence intensity indicating SSB and the mean fluorescence intensity per cell expressed in arbitrary units are shown in panels (**C**) and (**D**), respectively.

Next, we wanted to know whether the halophilic archaea might exacerbate the genotoxic effects of SEB or whether it could prevent it. Therefore, DCs were incubated with *Hrd. rudnickae* or *Nnm. salaciae* and then stimulated with SEB. The degree of chromatin condensation, the percentage of chromatin fragmentation, as well as the SSB and DSB of DCs were assessed. The negative control was unstimulated DCs, and the positive control was SEB-stimulated DCs without prior incubation with the halophiles. As expected from the above results, after 24 h of stimulation, no significant differences were observed in the degrees of chromatin condensation in any condition, compared to unstimulated cells (Fig. 6A). In contrast, chromatin fragmentation within DCs, significantly increased in SEB-treated compared to unstimulated cells (*p* = 0.0286) (Fig. 6B). Interestingly, in DCs treated with either one of the two halophilic archaea before stimulation with SEB, the degree of chromatin fragmentation was significantly lower compared to SEB-stimulated cells without prior incubation with the halophiles (*p* = 0.0286 for SEB vs. SEB + *Hrd. rudnickae* and *p* = 0.0095 for SEB vs. SEB + *Nnm. salaciae*) (Fig. 6B).

As shown above, SEB stimulation of DCs resulted in a significantly increased percentage of DCs cells with SSB (Fig. 6C) (*p* = 0.0286) and DSB (Fig. 6E) (*p* = 0.0286) and in a significantly increased number of SSB (Fig. 6.D) (*p* < 0.0001) and DSB (Fig. 6F) (*p* < 0.0001) per cell compared to unstimulated cells. Again, incubation of the DCs with *Hrd. rudnickae* or *Nnm. salaciae* before stimulation with SEB resulted in a significant reduction in the percentage of DCs with SSB (Fig. 6C) (*p* = 0.0286 and *p* = 0.0286, respectively) and DSB (Fig. 6E) (*p* = 0.0286 and *p* = 0.0286, respectively), as well as in the amount of SSB (Fig. 6D) (*p* = 0.0006 and *p* < 0.0001, respectively) and DSB (Fig. 6F) (*p* < 0.0001 and *p* < 0.0001, respectively) per cell, compared to DC stimulated with SEB only.

### Halophilic archaea restore normal cell cycle and reduce apoptosis in SEB-stimulated DCs

The protective effects of the two halophilic archaea against SEB-mediated cytotoxicity prompted us to investigate their potential protective effect on SEB-induced cell-cycle disturbances, apoptosis or necrosis of DCs.

**Fig. 7:**
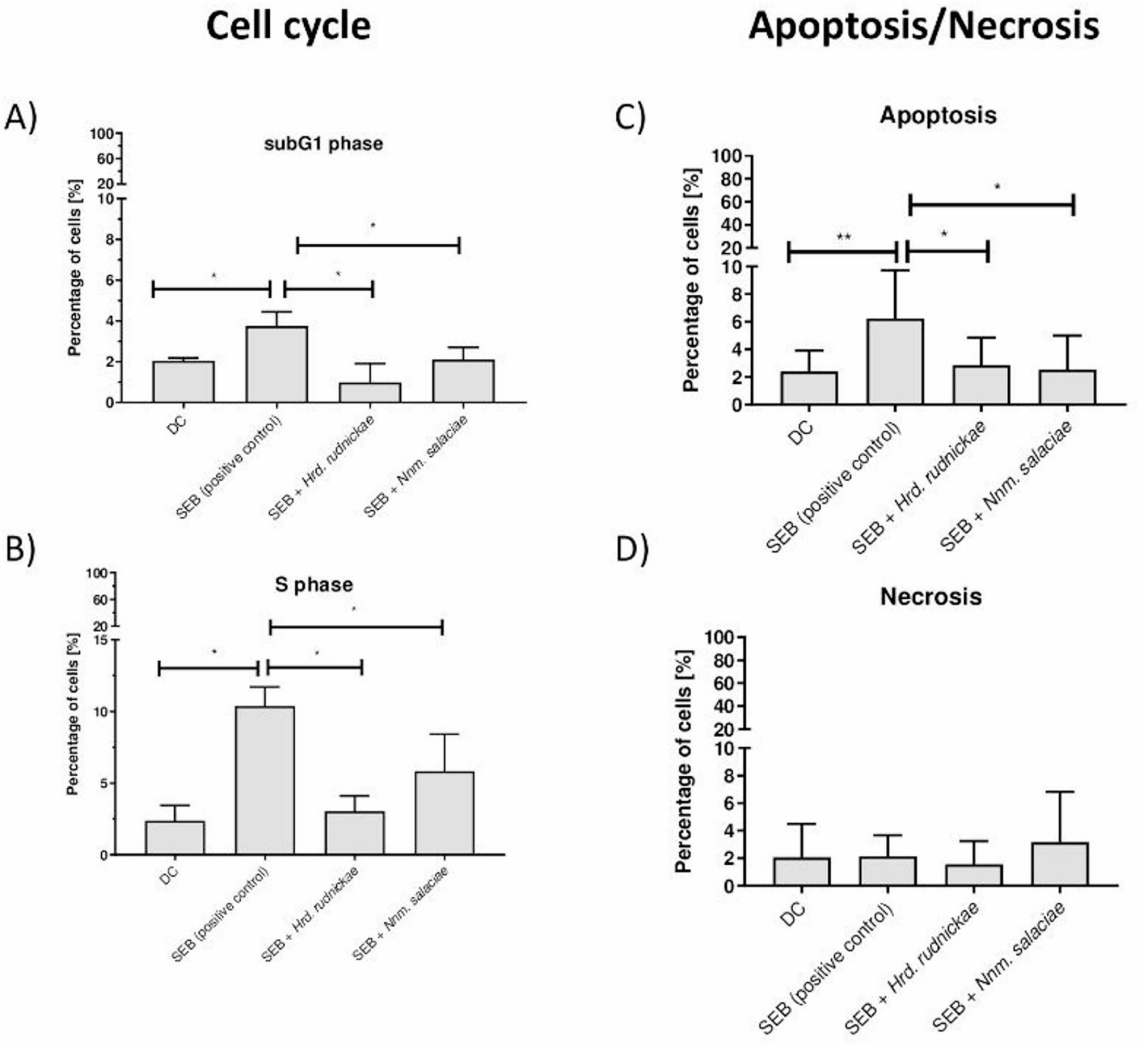
Protection by halophilic archaea against SEB-induced cell-cycle disturbances and apoptosis. Percentages of unstimulated DCs, used as negative controls, DCs incubated with *Hrd. rudnickae* or *Nnm. salaciae* in the presence of SEB or DCs stimulated with SEB alone in the cell cycle subG1 (**A**), and S phases (**B**) were assessed by flow cytometry. Percentages of unstimulated DCs, DCs incubated with *Hrd. rudnickae* or *Nnm. salaciae* in the presence of SEB and DCs stimulated with SEB alone undergoing apoptosis and necrosis are shown in panels (**C**) and (**D**), respectively. Data are presented as means ± standard deviation based on 7 independent experiments. *p ≤ 0.05; **p ≤ 0.01.

As seen above, SEB-stimulated DC showed a significant increase in the percentages of cells in the sub-G1 (*p* = 0.0286) (Fig. 7A) and S phases (*p* = 0.0286) (Fig. 7B). The percentages of DCs treated with the halophilic archaea before stimulation with SEB was significantly lower in the sub-G1 (*p* = 0.0286) and S phases (*p* = 0.0286) compared to cells stimulated with SEB alone (Fig. 7A and B).

Similarly, while the percentage of apoptotic DCs increased upon SEB treatment, compared to non-stimulated cells (*p* = 0.0062) (Fig. 7C), incubation of the cells with *Hrd. rudnickae* or *Nnm. salaciae* 24 h prior to SEB stimulation significantly decreased the percentage of DCs in the apoptotic state compared to DCs stimulated with SEB alone (*p* = 0.0365 for SEB vs. SEB + *Hrd. rudnickae* and *p* = 0.0152 for SEB vs. SEB + *Nnm. salaciae*) (Fig. 7C). As expected from the above results, either SEB alone, nor SEB in the presence of *Hrd. rudnickae* or *Nnm. salaciae* induced significant necrosis of the Mo-DCs (Fig. 7D).

## Discussion

Although the interaction of pathogenic and commensal microorganisms with DCs has been intensively studied, little is known about the interactions of environmental microorganisms, especially of extremophilic archaea, with the human immune system. In our previous paper, we showed that human DCs recognize halophilic archaea and induce the production of cytokines (IL-10, IL-12p40, TNF-alpha)^7^. A similar effect was observed in DCs stimulated with methanogenic archaea present in the human gut^5,6^. Here, we extended our previous study and show for the first time that environmental halophilic archaea can enter human Mo-DCs. Previous studies have shown that intestinal methanogens like *M. stadtmanae* are rapidly phagocytosed by human DCs^6^, but were not taken up by Caco-2 colon epithelial cells^6^. Here, we used fluorescence microscopy to show that two strains of halophilic archaea *Hrd. rudnickae* and *Nnm. salaciae* reside in abundant amounts within the cytoplasm and the nucleus of human DCs and validated these observations independently by PCR amplification of the 16 S rRNA gene of the archaea residing within the DCs. However, it is possible that survival of these halophilic archaea within the DC nucleus and cytoplasm is of limited duration, as we have not been able to culture them after 24 h of uptake by the DCs.

Invasion of bacteria into host cells was shown to modify gene expression and cell proliferation via interactions with cytoplasmic components of the infected cells. Furthermore, it was shown that bacterial factors can act directly within the nucleus and thereby induce permanent genetic or long-term epigenetic effects on the host cell^12,13^. As many cell-invading bacteria cause DNA damage, such as SSB and DSB, it was of importance to know whether the halophilic archaea that can invade DCs may induce DNA damage, which may lead to apoptosis, or secondarily to cell necrosis^14^. Microorganisms, such as *Pseudomonas aeruginosa*, *Chlamydia trachomatis*, *Haemophilus ducreyi*, *Escherichia coli*, *Helicobacter pylori* or their components, including Exotoxin S of *P. aeruginosa* and the cytolethal distending toxin produced by *E. coli* or *H. ducreyi*^15^, *Mycobacterium bovis* and *M. bovis* BCG^16^ can cause chromosomal DNA fragmentation in DCs. However, neither chromosomal DNA fragmentation nor DNA SSB or DSB were observed after DCs were incubated with *Hrd. rudnickae* or *Nnm. salaciae*. Consequently, neither of these two halophilic archaea induced cell apoptosis or necrosis.

Cell cycle inhibition is also one of the ways by which pathogens interact with host cells, as has been shown for pathogenic bacteria, such as *E. coli*, *Campylobacter* spp., *Mycobacterium ulcerans* or *Yersinia pseudotuberculosis*, which use cyclomodulins to inhibit the cell cycle of B lymphocytes, T lymphocytes, fibroblasts, macrophages or epithelial cells^17^. Bacterial cyclomodulins^18^ have been shown to act within the nucleus, where they use two main strategies: deregulation of cell-cycle-specific proteolysis and induction of DNA damage. As such, the cyclomodulin cytolethal distending toxin prevents the cell cycle transition from the G2 to the M phase, resulting in the inhibition of mitosis and cell proliferation^17^, The cell cycle regulates many processes, including the proliferation of immune cells and cellular differentiation. The cyclomodulin FIP, produced by *Fusobacterium nucleatum*, causes the accumulation of B and T lymphocytes in the G1 phase, thereby impairing the cellular response to antigens^17^. It has also been shown that cyclomodulins enhance pathogen adhesion to host cells by inducing epithelial cell apoptosis, inhibiting T lymphocyte proliferation, reducing defensin production or inhibiting protein synthesis^19^. We were therefore interested to see whether halophilic archaea may affect the cell cycle of DCs and found that the halophilic archaea *Hrd. rudnickae* and *Nnm. salaciae* did not affect the DC cell cycle at any phase, in contrast to SEB, a bacterial toxin produced by *Staphylococcus aureus*, used as a positive control, which increased the percentages of cells in the sub-G1 and S phases.

For many years it was believed that Mo-DCs did not proliferate and did not enter the cell cycle^20,21^. However, Cao et al. have shown that Mo-DCs from different age groups can enter the cell cycle^22^. The distribution of cells in the G1, G2 and S phases of human DCs described by Cao et al.^22^ was similar to those obtained in this study in which unstimulated DCs from healthy blood donors were analyzed, and it was not modified upon stimulation of the Mo-DCs with *Hrd. rudnickae* or *Nnm. salaciae*. These halophiles also did not induce apoptosis or necrosis of DCs. These observations indicate that these environmental microorganisms can be regarded as safe stimulators of human DCs.

Surprisingly, we found that both halophilic archaea protected against the genotoxic effects induced in DCs by SEB, which targets the nucleus and causes cell-cycle arrest by triggering DNA damage via mechanisms based on reactive oxygen species^23^. SEB has been reported to increase DNA fragmentation in mouse lymphocytes^24,25^, indicating SEB-induced DNA breaks. In this study, we show that SEB-stimulated Mo-DCs exhibited a significantly higher number of SSBs and DSBs per cell, compared to unstimulated DCs, but that incubation of Mo-DCs with *Hrd. rudnickae* or *Nnm. salaciae*, prior to SEB stimulation, resulted in a significant reduction of SEB-induced SSBs and DSBs.

Perrin et al. showed that SEB induces changes in the cell cycle of human PBMCs and CD4^+^ T cells, with an increase in the percentage of cells in the S and G2 phases and a decrease in the percentage of cells in the G1 phase^26^. Moreover, SEB-stimulated splenocytes from C57BL/6 mice also increased the percentages of cells in the S phase^27^. Our study shows that stimulation of human Mo-DCs with SEB resulted in a significant cell accumulation in the sub-G1 and S phases. However, when the cells were incubated with the halophilic archaea prior to SEB stimulation, a significant reduction in cell accumulation in these phases was observed. As a strong superantigen, SEB is also known to induce apoptosis in a variety of cell types, including THP-1^28^, ECV304^29^, human monocytes^30^ and V_β_8^+^ T cells^31,32^. In the present study, we observed that Mo-DCs stimulated with 1 µg/mL SEB showed a significantly increased percentage of apoptosis compared to unstimulated cells, likely through caspase-8 activation^29,33^, although this was not investigated here. In contrast, when the Mo-DCs were incubated with the halophilic archaea before the addition of SEB, a significant decrease in the percentage of apoptotic cells was observed, indicating a strong protective effect of the halophiles against SEB-induced genotoxic effects in DCs.

The mechanism by which these halophilic archaea protect cells against the genotoxic effects of SEB and whether this mechanism requires the presence of the halophiles within the nucleus and/or the cytoplasm remains to be investigated. However, as it has been shown that extremophilic archaea can produce compatible compounds, such as betaine, ectoine, and glycine, polyols (arabitol, glycerol, and sorbitol), and sugar derivatives (glucosylglycerol, sucrose, and trehalose), it is possible that such compounds may play a role in the protective effect^34,35^. We have previously reported that both halophilic archaea secrete potent anti-cancer compounds^36^. Whether these play a role in the protective effects against SEB cytotoxicity is also unknown. It may also be possible that the two halophilic species exert their protective effects via distinct mechanisms, as they are members of different genera. Finally, it will be important to investigate whether the cell/nucleus invasion and the protective properties observed here can also be seen with other extremophilic archaea.

In conclusion, this study shows for the first time that the halophilic archaea *Hrd. rudnickae* and *Nnm. salaciae* can invade the cytoplasm and nucleus of human Mo-DCs, but do not induce any significant harmful effect on DNA structure and DC cell cycle, and do not induce apoptosis or necrosis of human DCs. In conjunction with our previous observations showing potent stimulation of DCs^7^ by these halophilic archaea thus make them safe simulators for the immune system. Moreover, SEB-induced genotoxic effects in DCs, manifested by the presence of SSB and DSB in the DNA, changes in cell cycle and the induction of apoptosis, were significantly reduced when the DCs were incubated in the presence of these halophilic archaea. In addition to SEB, many other to physical and chemical agents, such as ionising radiation, etoposide, epirubicin^37^, cepharanthine^38^, xanthohumol^39^ and cladribine^40^, can cause chromosomal DNA fragmentation, which can lead to the development of a variety of diseases, including cancer, neurodegenerative diseases, infertility, immunodeficiencies or microcephaly^37^. Whether the halophilic archaea also prevent DNA fragmentation by these physical and chemical agents remains to be investigated. This would certainly illustrate the wide potential of environmental halophilic archaea as safe immunomodulators and protective agents for different disorders.

## Methods

### Ethics statement

The study was conducted according to the principles of the Declaration of Helsinki and was approved by the Ethics Committee at the University of Lodz, Poland (3/KBBN-UŁ/II/2017). Commercially available buffy coats were purchased from the Regional Blood Donation Station, Lodz, Poland. Written informed consent from all study subjects for using buffy coats for the research was obtained by Regional Blood Donation Station in Lodz before blood collection.

### Preparation of halophilic archaea

*Hrd. rudnickae 64* (DSM 29498^T^) and *Nnm. salaciae* (DSM 25055^T^) were kindly provided by Dr Luciana Albuquerque from the Center for Neuroscience and Cell Biology Biotech, Biocant Park, Cantanhede, Portugal and Prof. Milton S. da Costa from the University of Coimbra, Portugal. The halophilic strains were cultivated in *Halobacteria* medium (HBM) as described^7^. For DC stimulation, archaea from 48 h cultures at logarithmic growth were harvested according to the protocol described by Krawczyk et al. 2022^7^.

### Peripheral blood mononuclear cell isolation

Peripheral blood mononuclear cells (PBMCs) were isolated from seven anonymized, commercially available buffy coats of healthy human blood donors obtained from the Regional Blood Donation Station in Lodz, Poland. The cells were prepared according to the protocol described previously^7^. Briefly, the cells were diluted 1:1 in PBS and layered on Ficoll-Paque PLUS. After centrifugation PBMCs were resuspended in RPMI-1640 supplemented with 100 U/ml penicillin, 0.1 mg/ml streptomycin, 2 mM L-glutamine (Gibco, Grand Island, NY) and supplemented with 10% (v/v) heat-inactivated FCS (Cambrex, Belgium) (complete RPMI-1640 (cRPMI-1640))^7^. CD14^+^ human monocytes were purified from PBMCs by positive immunomagnetic separation using anti-human CD14^+^ MACS Microbeads (Miltenyi Biotech, Germany) as described^41^.

### Generation of Mo-DCs

The percentage of monocytes, obtained from PBMCs by positive immunomagnetic separation method was 23.41% ± 3.30%. Monocytes were suspended in cRPMI-1640, seeded into 6-well flat-bottomed plates (Falcon) at the density 1 × 10^6^ cells/mL and cultured for 6 days at 37 °C, 5% CO_2_ in the presence of 10 ng/ml IL-4 and 25 ng/ml GM-CSF (R&D Systems, Minneapolis, MN) to allow the cells to differentiate into immature Mo-DCs. After 6 days of culture, the cells were harvested, pooled and counted. The mean percentages of Mo-DCs obtained by this method were 55.43% ± 10.77%. Cell debris that might have occurred by cell lysis were excluded by the gating strategy based on changes in the intensity of light scattering parameters - forward scatter channel (FSC-A) and side scatter channel (SSC-A) (Fig. S6A). Doublets were excluded by placing the gates on a plot of height (H) or width (W) versus area (A). for the forward scatter channel (FSC) or side scatter channel (SSC). Doublets would have double values of the area and width values of single cells, while the height would be similar (Fig. S6B and C).

### Stimulation of immature Mo-DCs

Immature Mo-DCs at a density of 1 × 10^6^ cells/mL were incubated for 24 h (37 °C, 5% CO_2_) with 1 × 10^6^ cells of *Hrd. rudnickae*, *Nnm. salaciae* or SEB (Sigma-Aldrich 1 µg/ml), or with *Hrd. rudnickae* and *Nnm. salaciae* in the presence of SEB. Mo-DCs in cRPMI-1640 medium was used as the negative control.

### Presence of halophile archaea within Mo-DCs

To identify halophilic archaea within Mo-DCs, the AO/EB staining method was used according to Rybaczek et al.^10^. DCs were stimulated with halophilic archaea for 24 h and then stained with 1 ml of 100 µg/ml AO, 100 µg/ml EB in PBS for 4 min. The Mo-DCs were then washed twice with PBS and fixed with 1% glutaraldehyde in PBS for 15 min.

The cells were then washed twice with PBS, placed on SuperFrost Plus slides and examined using an Optiphot-2 fluorescence microscope (Nikon) equipped with a B-2 A filter (blue light; λ = 495 nm). Images were captured using a DS-Fi1 microscope camera (Nikon) and Act-1 software (Precoptic, Warsaw, Poland). DC images with halophilic archaea were analyzed by using ImageJ v1.37c software (Public Domain by Wayne Rasband).

### PCR amplification

DCs were incubated at a density of 1 × 10^6^ cells/mL for 24 h at 37°C and 5% CO_2_ with 1 × 10^6^ cells halophilic archaea *Hrd. rudnickae*, *Nnm. salaciae* or remained unstimulated (negative control). After 24 h of incubation DCs were collected from 24-well plates and washed three times using cold, isotonic Phosphate-Buffered Saline (PBS), each time centrifuged at 21°C for 10 min. at 160x*g*. Finally, the pellets were frozen at -20°C until further treatment. The pellets of DCs were thawed at 21°C, lysed with 200 µl 1% Triton X-100 in water and vortexed intensively for 30 sec. The samples were then incubated for 30 min. at 4°C and vortexed every 10 min. Finally, the DCs were heated for 10 min. at 70°C, and the lysates were stored at − 20°C. The 16S rRNA gene was amplified by PCR using the archaea-specific primers 21F (5’-TTCCGGTTGATCCTGCCGGA-3’) and 1492R (5’-TACGGYTACCTTGTTACG-3’)^42^, Polymerase Taq (NZYtech) Mastermix and the stored cell lysates at the total volume of 50 µl. The following PCR protocol was used for the amplification: 94 °C 5 min., 1 cycle; 94 °C 1 min.; 55 °C 0.5 min., 72 °C 2 min., 40 cycles; 72 °C 10 min., 1 cycle; 4 °C. The 16 S rRNA PCR products were loaded onto a 2% agarose gel in 1x TAE buffer (Merck) and 7 µl of EB. The electrophoresis was run with a 5 v/cm (50 V) current for 1.5 h. The DNA ladder (1000 bp) (Thermofisher) was used as a size marker.

### Mitotic and aberration indexes

Mo-DCs were incubated with halophilic archaea, SEB or halophilic archaea in the presence of SEB and then placed on ThermoScientific™ Nunc™ Lab-Tek™ ChamberSlide™ culture slides and incubated for 24 h. Mo-DCs were fixed with 4% paraformaldehyde in PBS for 15 min. and then incubated for 1 h with 10% horse serum, 1% BSA, 0.02% sodium azide in PBS at room temperature to minimize the risk of non-specific antibody binding. Mo-DCs were then incubated overnight in a humidified chamber (4 °C) with a rabbit anti-human β-tubulin antibody (Sigma-Aldrich). Cells were washed three times with PBS/0.2% Triton X-100 and then incubated for 1 h in the dark at room temperature with goat anti-rabbit antibodies (Cell Signaling Technology) conjugated with Alexa Fluor 488. Mo-DCs were then washed three times with PBS/0.2% Triton X-100 and once with PBS, followed by staining for 5 min. with DAPI (0.1 mg/mL). The cells were examined by using an Axio.Imager.A1 fluorescence microscope equipped with a B-2 A filter (λ = 450–490 nm) for AlexaFluor 488 and a UV-2 A filter (UV light; λ = 518 nm). The chromatin fragmentation percentage, also known as the aberration index, was quantitatively measured by assessing DAPI fluorescence. When measuring the fluorescence of the entire cell nucleus, a lower result indicates chromatin fragmentation. Additionally, the mitotic index, which represents the percentage of cells undergoing mitosis, was also measured. Quantitative analyses of Mo-DCs images were performed by measuring the fluorescence level separately for DAPI and AlexaFluor 488 fluorochrome (β-tubulin conjugated) in individual cells using ImageJ software. The results were visualized using Photoshop CS5.

### Single and double strand DNA break

Mo-DCs were incubated with halophilic archaea, SEB or halophilic archaea in the presence of SEB for 24 h and then placed on ThermoScientific™ Nunc™ Lab-Tek™ ChamberSlide™ culture slides and incubated for 24 h at 37 °C in 5% CO_2_. For detection of the SSB marker poly(ADP-ribose)-2 polymerase (PARP-2; EC 2.4.2.30) and of the DSB marker phospho-H2AX (Ser139), β-tubulin and actin (stained with rhodamine-labelled phalloidin), Mo-DCs were fixed for 15 min. with 4% paraformaldehyde in PBS and then incubated for 1 h with 10% horse serum, 1% BSA, 0.02% sodium azide in PBS at room temperature. Mo-DCs were then incubated with PARP-2, phospho-H2AX, rabbit anti β-tubulin antibodies and rhodamine-labelled phalloidin overnight in a humid chamber at 4 °C. DCs were then washed three times with PBS/0.2% Triton X-100 and incubated for 1 h in the dark at room temperature with goat anti-rabbit antibodies conjugated with AlexaFluor 488 (for β-tubulin and PARP-2) and goat anti-rabbit antibodies conjugated with AlexaFluor 555 (for phospho-H2AX). Mo-DCs were then washed three times with PBS/0.2% Triton X-100 and once with PBS. DNA in Mo-DCs was detected by staining with 0.1 mg/ml DAPI (for PARP-2) or 0.2 µg/ml propidium iodide (for phospho-H2AX) for 5 min. in the dark. The cells were then examined by using an Axio.Imager.A1 fluorescence microscope equipped with a B-2 A filter (λ = 450–490 nm) for AlexaFluor 488 and a G-2 A filter (λ = 518 nm) for AlexaFluor 555 and propidium iodide. Quantitative analyses of Mo-DCs images were performed by measuring the fluorescence level separately for DAPI and propidium iodide (for DNA) and the fluorochromes AlexaFluor 488 (for β-tubulin and PARP-2) and AlexaFluor 555 (for phospho-H2AX) in individual cells by using ImageJ software. Visualization of the results was performed using Photoshop CS5.

### Cell cycle analysis

After incubation of Mo-DCs with halophilic archaea, SEB or halophilic archaea in the presence of SEB, 1 ml of total culture supernatants were collected and 1 ml of cold EDTA was added to the cells and incubated for 10 min. at 4 °C. Cells were harvested by centrifugation at 350 x *g* for 10 min., at 4 °C. The pellets were resuspended in 1 ml cold PBS and centrifuged again (350 x *g*, 10 min., 4 °C). The cells were resuspended in 100 µl of cold PBS and transferred to a new vial containing 1 ml of ice-cold 70% ethanol. The sediment of cells was resuspended in 0.5 ml solution A (75 µmol/dm^3^ propidium iodide (PI) and 50 IU Kunitz/ml DNase-free RNase in PBS) (Sigma-Aldrich) for 30 min. at 37 °C. Measurements were performed using a BD LSRII flow cytometer at the Cytometry Laboratory of the Faculty of Biosciences, University of Lodz. The percentages of cells in subG1, G1, S and G2 phases of the cell cycle were determined by using FlowJo analysis software.

### Apoptosis/necrosis detection

The fluorescein isothiocyanate (FITC) Annexin V Apoptosis Detection Kit (Becton Dickinson, San Jose, CA, USA) was used to differentiate between apoptotic and necrotic cells. To determine the percentage of Mo-DCs undergoing apoptosis or necrosis, stimulated and unstimulated cells were collected and washed with cold PBS. Mo-DCs were then resuspended in 100 µl of 1% annexin buffer (BD Pharmingen). 5 µl of FITC and 5 µl propidium iodide were then added to each vial, and the cells were incubated for 15 min. at room temperature in the dark. Measurements were performed by using a BD LSRII flow cytometer in the Cytometry Laboratory of the Faculty of Biology and Environmental Protection, University of Lodz.

### Statistical analysis

Statistical analyses were performed with the GraphPad Prism 7 and STATISTICA 12.0 PL program. Data are expressed as mean ± standard deviation. After verifying assumptions assays, including testing normality by the Kolmogorov-Smirnov test, the type of data and the number of data, the non-parametric statistics were used. Differences between samples were analyzed by Kruskal-Wallis test and Mann-Whitney U test (for impaired data). *p* values ≤ 0.05 were considered significant.

## Electronic supplementary material

Below is the link to the electronic supplementary material.

Supplementary Material 1

## Data Availability

The data that support the findings of this study are available from the corresponding author upon reasonable request.
