# Supplementary material for: Uptake of environmental halophilic archaea by human dendritic cells
Source: Sci Rep. 2025 Jul 1;15:21912. doi: 10.1038/s41598-025-07365-z (PMC12217356; doi:10.1038/s41598-025-07365-z)
Supplement: Supplementary file 1 — Supplementary Material 1 [file 41598_2025_7365_MOESM1_ESM.pdf]

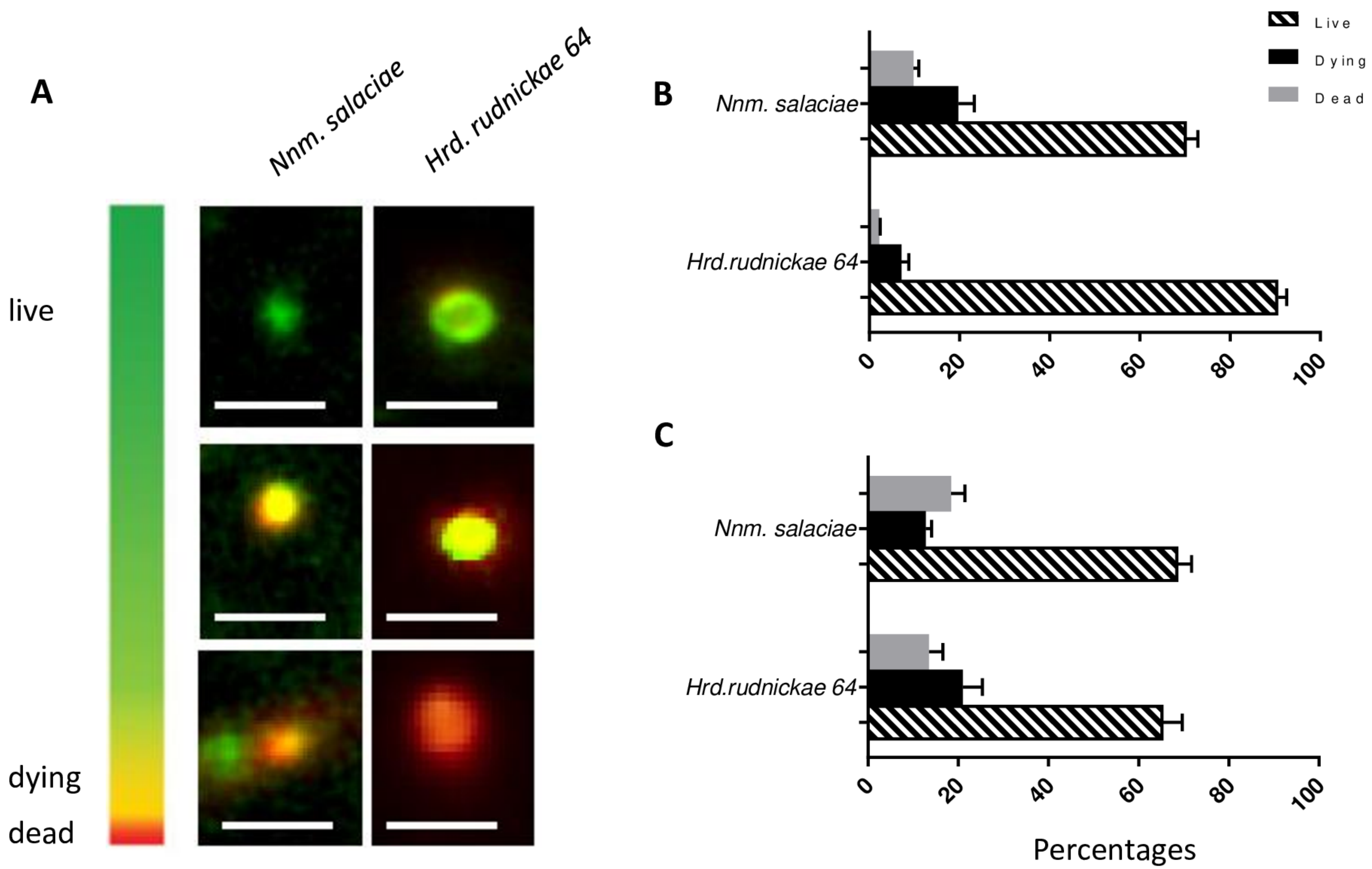

Fig. S1

Evaluation of dead, dying and living *Hrd. rudnickae* 64 and *Nnm. salaciae* by double staining with acridine orange and ethidium bromide. (A) Representative fluorescence micrographs of living (green), dying (yellow-to-orange) and dead (red) *Hrd. rudnickae* 64 (left) and *Nnm. salaciae* (right) incubated in RPMI/10%FCS culture medium. (B and C) Percentages of living (hatched bars), dying (black bars) and dead (grey bars) *Hrd. rudnickae* 64. and *Nnm. salaciae* after incubation for 1h (B) and 30 days (C) in RPMI/10%FCS culture medium based on the color spectrum. The data are shown as means ± SD from three independent experiments. Scale bar=10µm.

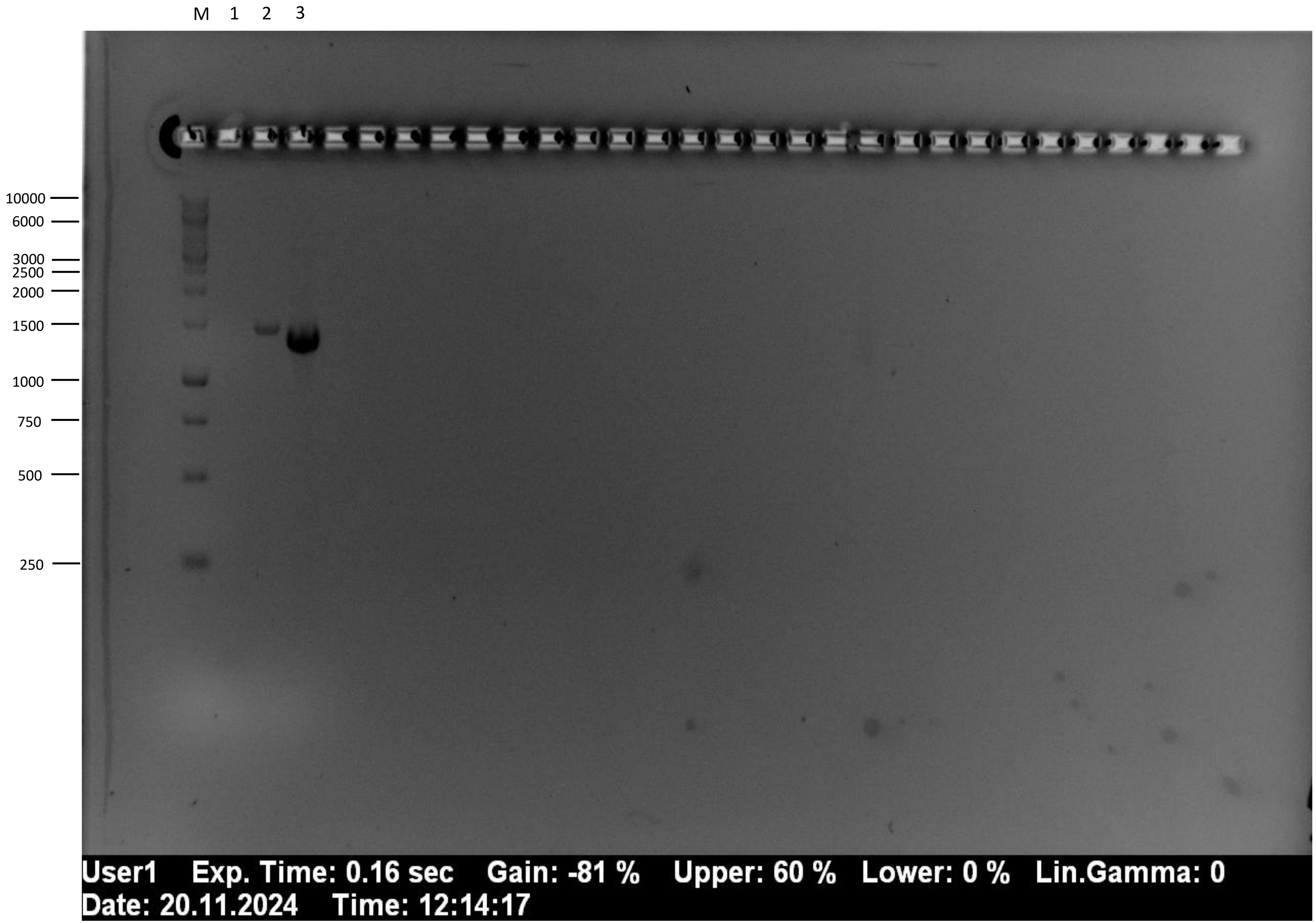

Fig. S2. Original agarose gel electrophoresis of the amplicons corresponding to the 16S rRNA gene in the non-stimulated DC (lane 1), DC incubated with *Nnm. salaciae* (lane 2) or *Hrd. rudnickae* (lane 3). Electrophoresis was performed on a 2% agarose gel and run with a 5v/cm current for 1.5 h. Lane M shows the size markers and the left margin the numbers of base pairs.

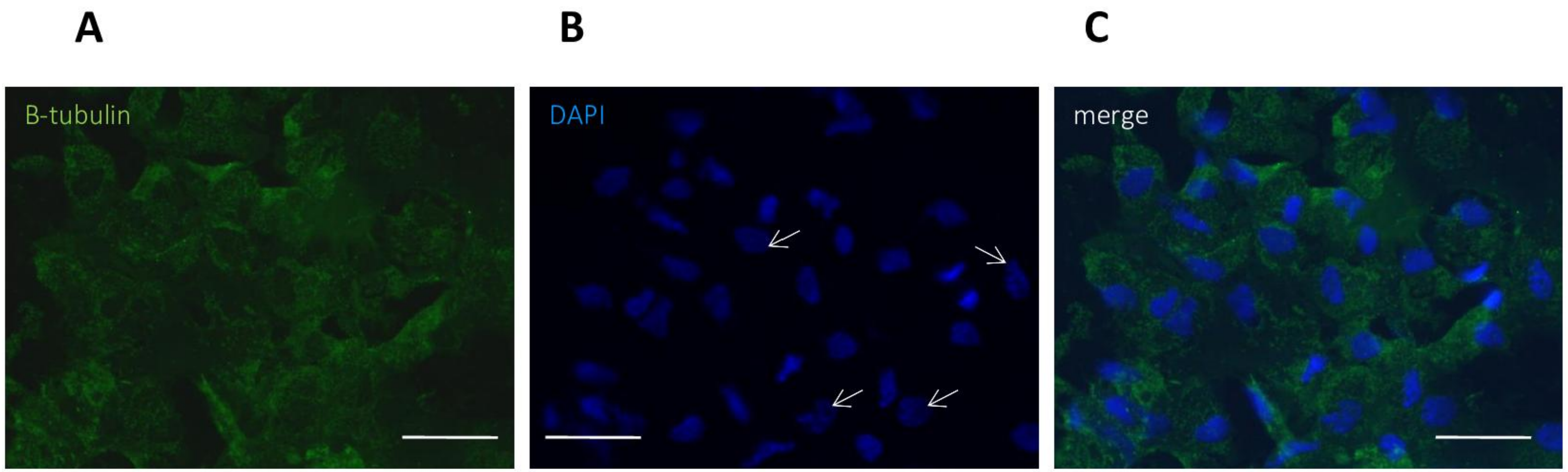

Fig. S3

Fluorescence microscopy images of untreated dendritic cells stained with monoclonal antibodies against β-tubulin (A) and DAPI (B). C shows the merged images (β-tubulin+DAPI). Scale bar=10μm

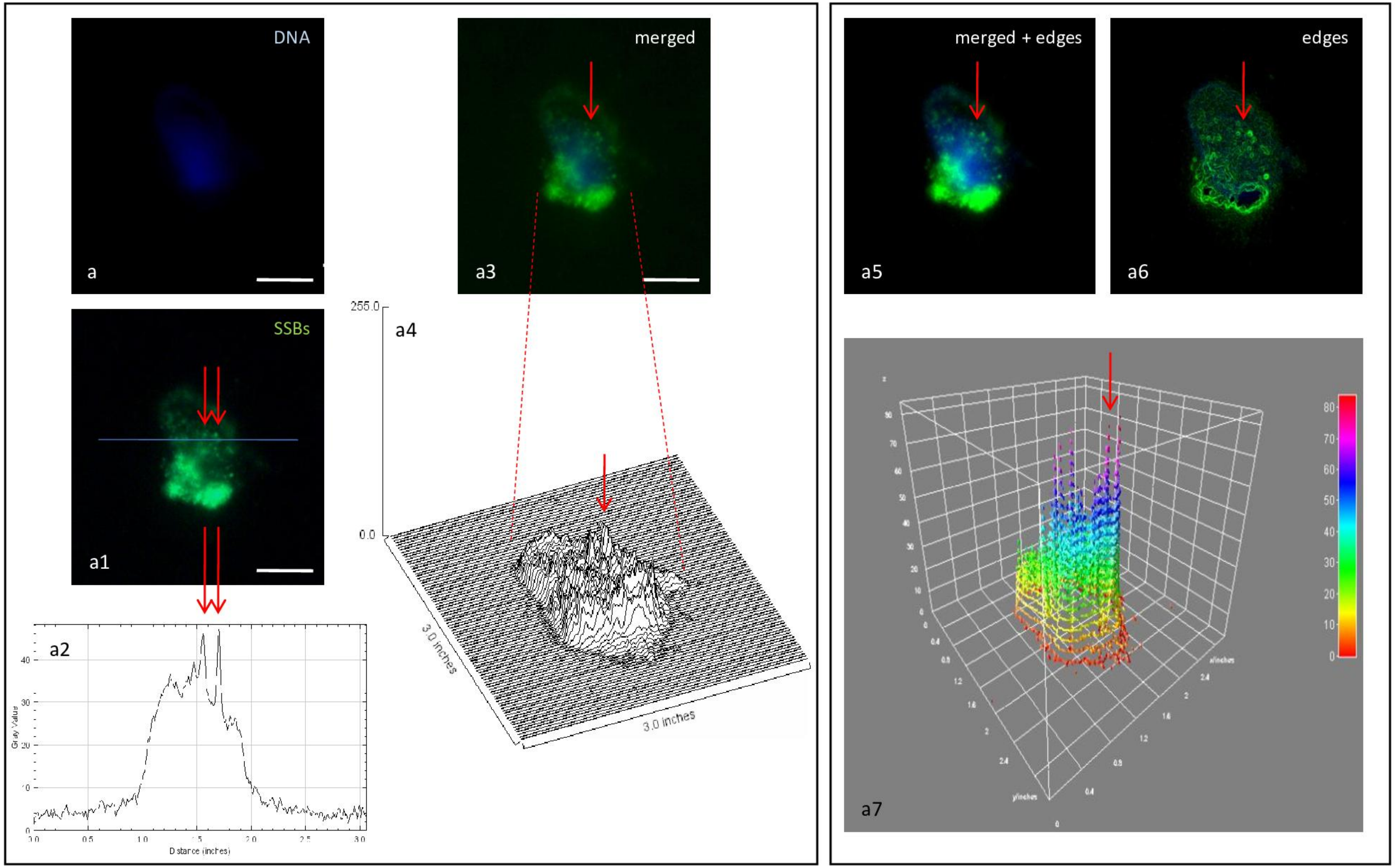

Fig. S4

Single-stranded DNA breaks in dendritic cells treated with *Staphylococcus* enterotoxin B. DNA was stained with DAPI (a) and single-stranded DNA breaks (SSBs) with an anti-Poly(ADP-ribose)-2 polymerase antibody (a1) after treatment of dendritic cells with *Staphylococcus* enterotoxin. SSBs were quantified using the ImageJ software and shown in a histogram indicating the enhanced fluorescence at the sites of SSBs (a2). Merged image is shown in a3, and a4 shows the spatial structure demonstrating an increase in fluorescence at the site of SSBs. Red arrows indicate the foci of the SSB. a5 and a6 depict the edges that are sites of enhanced fluorescence indicating the SSBs, and a7 depicts the spatial structure of the dendritic cell nucleus displaying enhanced fluorescence indicating the SSBs. The scale bar = 10µm

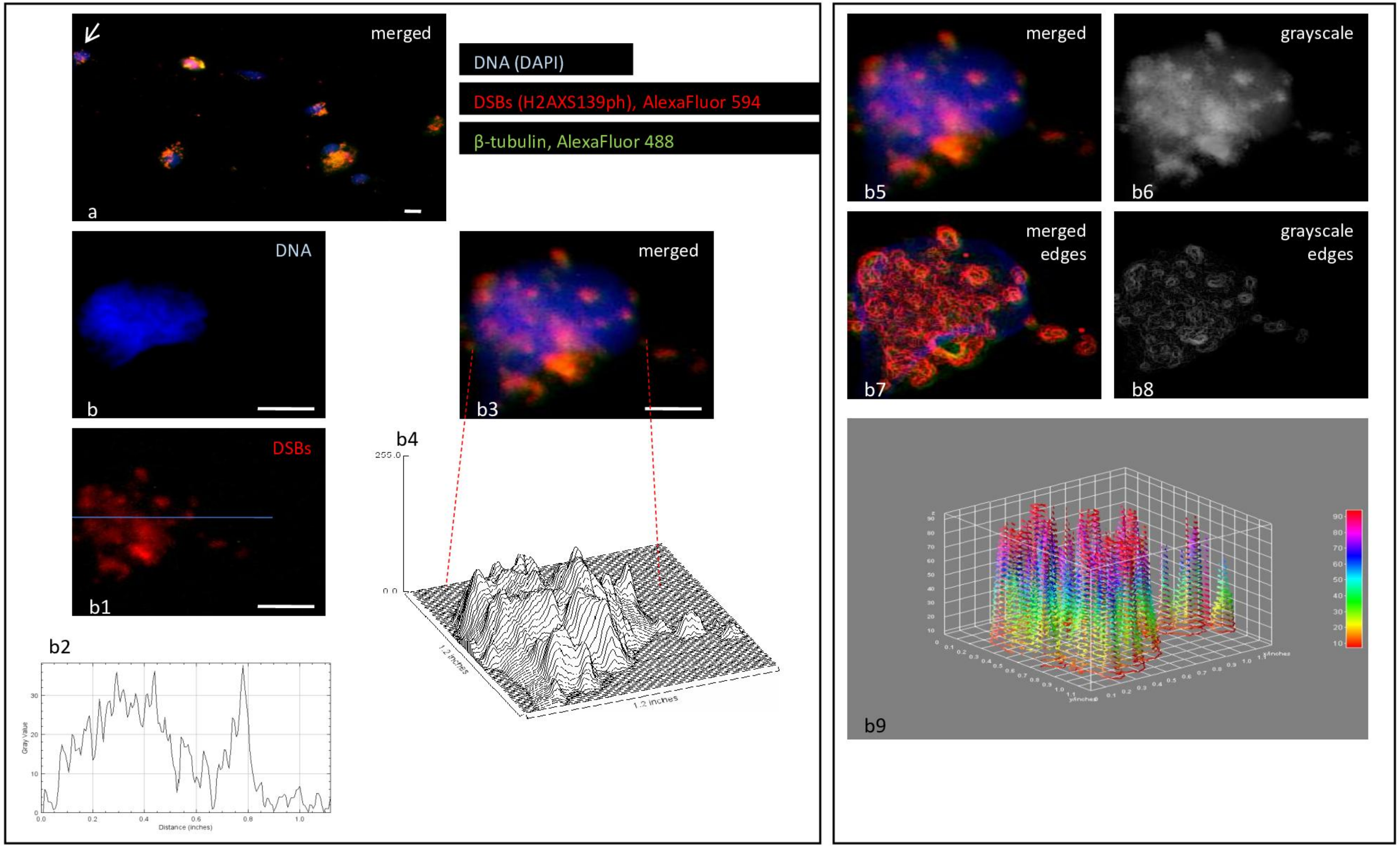

Fig. S5

Double-stranded DNA breaks in dendritic cells treated with *Staphylococcus* enterotoxin B. DNA was stained with DAPI (a and b) and double-stranded DNA breaks (DSBs) with an anti-phospho-H2AX (Ser139) histone antibody (b1) after treatment of dendritic cells with *Staphylococcus* enterotoxin. a and b3 show the merged image of DAPI, phospho-H2AX (Ser139) histone and tubulin staining. b2 shows the histogram indicating enhanced fluorescence at the site of DSBs, and b4 the special structure demonstrating an increase in fluorescence at the sites of DSBs. b5-b8 depict the edges indicating enhanced fluorescence at the DSB sites. b5 and b7 show the enhanced fluorescence indicating the DSBs, and b6 and b8 show the areas of enhanced fluorescence indicating the DSBs in grey. b9 depicts the spatial structure of the DC nucleus displaying enhanced fluorescence at the sites of DSBs. The scale bar = 10μm

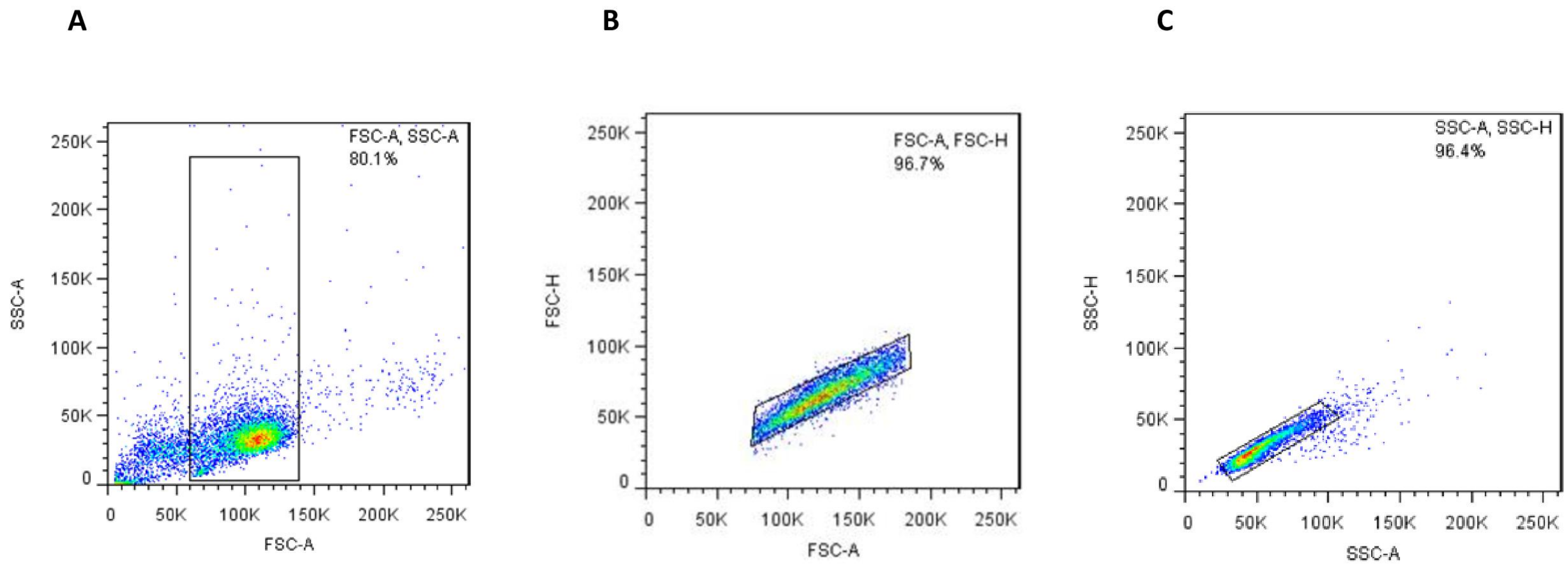

Fig. S6

Gating strategy for human dendritic cells. (A) Cell debris caused by lysis was excluded by gating using the forward scatter channel (FSC-A) and side scatter channel (SSC-A). Doublets were excluded by gating based on the height (FSC-H and SSC-H) and area (FSC-A and SSC-A) for the forward scatter channel (B) or side scatter channel (C).
